# Colonization behaviors of mountain pine beetle on novel hosts: Implications for range expansion into northeastern North America

**DOI:** 10.1371/journal.pone.0176269

**Published:** 2017-05-04

**Authors:** Derek W. Rosenberger, Robert C. Venette, Mitchell P. Maddox, Brian H. Aukema

**Affiliations:** 1 Department of Entomology, University of Minnesota, St. Paul, Minnesota, United States of America; 2 Department of Biological Sciences, Olivet Nazarene University, Bourbonnais, Illinois, United States of America; 3 United States Department of Agriculture—Forest Service, Northern Research Station, St. Paul, Minnesota, United States of America; 4 Chemistry Department, Bethel University, St. Paul, Minnesota, United States of America; Montana State University Bozeman, UNITED STATES

## Abstract

As climates change, thermal limits may no longer constrain some native herbivores within their historical ranges. The mountain pine beetle, *Dendroctonus ponderosae* Hopkins, is a tree-killing bark beetle native to western North America that is currently expanding its range. Continued eastward expansion through the newly invaded and novel jack pine (*Pinus banksiana* Lamb.) trees of the Canadian boreal forest could result in exposure of several species of novel potential host pines common in northeastern North America to this oligophagous herbivore. Due to the tightly co-evolved relationship between mountain pine beetle and western pine hosts, in which the insect utilizes the defensive chemistry of the host to stimulate mass attacks, we hypothesized that lack of co-evolutionary association would affect the host attraction and acceptance behaviors of this insect among novel hosts, particularly those with little known historical association with an aggressive stem-infesting insect. We studied how beetle behavior differed among the various stages of colonization on newly cut logs of four novel potential pine host species; jack, red (*P*. *resinosa* Ait.), eastern white (*P*. *strobus* L.) and Scots (*P*. *sylvestris* L.) pines, as well as two historical hosts, ponderosa (*P*. *ponderosa* Dougl. ex. Laws. var. *scopulorum* Engelm.) and lodgepole (*P*. *contorta* Dougl. var. *latifolia* Engelm.) pines. Overall, we found that beetle colonization behaviors at each stage in the colonization process differ between pine hosts, likely due to differing chemical and physical bark traits. Pines without co-evolved constitutive defenses against mountain pine beetle exhibited reduced amounts of defensive monoterpenoid chemicals; however, such patterns also reduced beetle attraction and colonization. Neither chemical nor physical defenses fully defended trees against the various stages of host procurement that can result in tree colonization and death.

## Introduction

In recent decades, human activity and climate change have contributed to the geographic range expansion of some herbivorous insects [1–4]. A number of forest insects have been highly successful in invading new areas [5] at high cost to the public [6–8]. Host shifts are one important factor that can mediate geographic range expansions [9], providing the invaders access to a new resource pool and/or corridor(s) for expansion. However, the ability to utilize new hosts is dependent upon a match between insect offensive and host defensive traits, or the “ecological fit” between herbivore and novel host [9,10].

The mountain pine beetle, *Dendroctonus ponderosae* Hopkins (Coleoptera, Curculionidae), is a bark beetle native to western North America ranging from southern California to British Columbia and east to the western edge of the Great Plains in western South Dakota. The beetle’s predominant hosts are lodgepole (*Pinus contorta* Dougl.) and ponderosa (*P*. *ponderosa* Dougl. ex. Laws.) pines, although the insect feeds and reproduces on almost all pines within its range [11]. This insect typically undergoes a one-year lifecycle, exhibiting a temperature-mediated synchronized emergence of adults in late summer crucial for host procurement activities [12,13]. Insect densities typically remain at low levels for decades, but populations can erupt when suitable host pools and environmental conditions coincide [4,14]. At outbreak levels, mountain pine beetles exhibit landscape-level effects on western North American pine forests [15], altering forest ecosystem services [16], forest regeneration [17], fire severity [18], carbon budgets [19–21], and even local climate [22].

**Fig 1:**
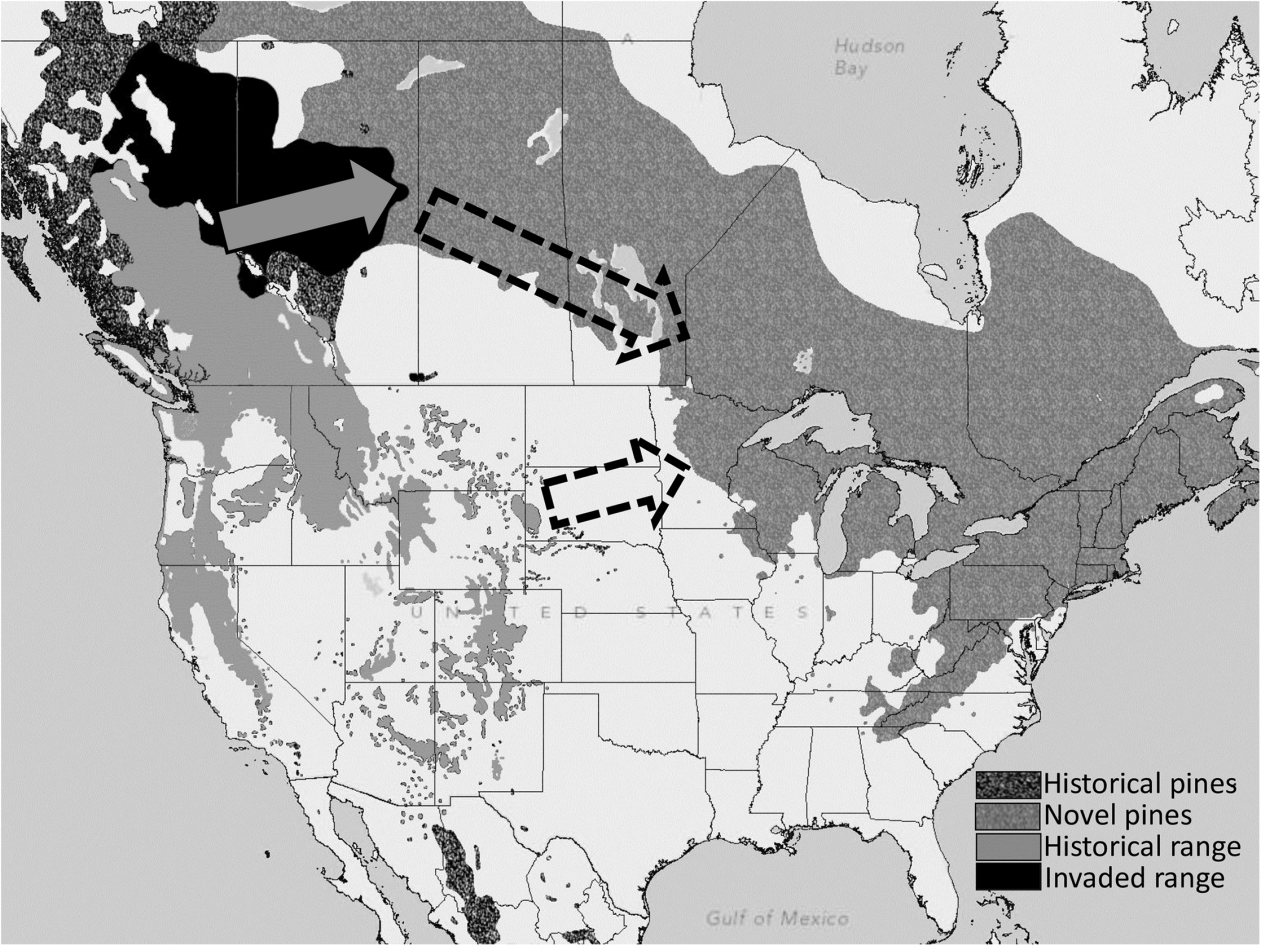
Approximate historical (light grey and mottled grey) and current (light grey and black) extent of mountain pine beetle range in North America. The light arrow represents current range expansion and dashed arrows represent potential pathways to eastern pine forests. Pine regions shown represent those of pine species used in this study from [33]. Historical and range expansion data obtain from data presented in [24] and the approximate geographic limit of beetle presence reported by the Alberta Ministry of Agriculture and Forestry in 2014.

Spread of mountain pine beetle to northeastern North America and its potential impact on forest and plantation trees such as red (*P*. *resinosa* Ait.), eastern white (*P*. *strobus*. L.), jack (*P*. *banksiana* Lamb) and Scots (*P*. *sylvestris* L.) pines are serious concerns [23,24]. To date, little is known about the ability of this insect to colonize these hosts. Two potential pathways could facilitate the introduction of mountain pine beetle to eastern forests (Fig 1). Anthropogenic movement of infested wood comprises the first pathway [1,2,25,26]. Similar anthropogenic introductions have been reported for other *Dendroctonus* spp. [27–29]. The second pathway reflects continued natural spread through the boreal forest [24]. In 2006, the beetle breached the geoclimatic barrier of the northern Rocky Mountains due to increased climatic suitability, and moved into lodgepole pine forests of western Alberta [30,31]. Over the past decade, populations of the insect expanded east into a lodgepole-jack pine hybrid zone and are now established and expanding though stands of pure jack pine, a “novel” host for this insect [32].

Similar to many bark beetles, the colonization of a susceptible tree by mountain pine beetle is characterized by a series of discrete events [34]. Female pioneers orient to the tree, find a bracing point on the bark, and determine the potential suitability of the tree from gustatory stimulant-deterrent cues in the bark and phloem [35,36]. If the host is accepted and boring commences, female mountain pine beetles produce an aggregation pheromone, *trans*-verbenol, from α-pinene, a monoterpene found in the trees’ phloem tissues [37]. Joining males produce additional aggregation pheromones [38], which enhance the signal of *trans*-verbenol in combination with several critical host volatiles [37,39–42]. This attraction fosters a mass attack that can concentrate low density populations [43] and quickly overwhelms the tree’s defenses [44].

Pines utilize several defensive mechanical and chemical traits such as bark texture, preformed resin ducts, and toxic chemical deterrents to curtail attacks [45], such that select trees with a higher defensive capacity may prevent colonization [46–48]. Various studies have reported differences in susceptibility to colonization among trees of a single species, concurrent with intraspecific variation in particular defensive traits [44,48–50]. However, greater variation in susceptibility can occur between trees of different species, where there are differences in both expression of defensive traits and the defensive strategy used (e.g., bark surface texture and internal tree chemistry) [41,46,50–53]. Many host defense studies have been conducted on live trees [51,52], however, constitutive defenses first encountered by the insects are also integral to mediating early colonization behaviors [53]. Interspecific differences in susceptibility to bark beetles have also been observed in harvested logs, for example [54–57]

Mountain pine beetle appears to have a long co-evolutionary history with western hosts, utilizing secondary chemicals produced by the tree to defend against insect and fungal attack [57,58] to instead produce aggregation pheromones and pheromone synergists that result in mass attack and host procurement [39]. Long associations may have resulted in particularly high secondary chemical concentrations in some hosts, which has conferred some level of resistance due to deterrence when concentrations of secondary chemicals are too high [36,59]. Thus, colonization behaviors mediated by defensive traits of a host may differ between historical hosts and novel host species. Empirically, mountain pine beetle has colonized novel host species on only two known occasions in arboreta, exhibiting varying degrees of success among species and between studies [60,61]. Thus, it is unclear if pine species with which the insect has had no co-evolutionary relationship will be suitable hosts[9].

To determine whether novel eastern pine hosts may be suitable hostsfor mountain pine beetle, and/or whether they have traits that may limit colonization, we designed a series of field and laboratory behavioral experiments to assess beetle response to novel pine hosts at each stage of the colonization process (attraction to infested material, bark acceptance, phloem acceptance, and egg gallery establishment). We used two common historical hosts, ponderosa and lodgepole pine, as positive controls to account for potential interspecific variation among historical hosts [54]. We hypothesized that interspecific differences among pine species will mediate host colonization behavior.

## Materials and methods

### Host material

We tested eastern white pine, jack pine, and red pine, all native to northeastern North America and of uncertain host status for mountain pine beetle; Scots pine, a Eurasian species commonly planted in North America and also of unclear host status, and ponderosa (*P*. *ponderosa* Dougl. ex. Laws. var. *scopulorum* Engelm.) and lodgepole pine (*P*. *contorta* var. *latifolia* Dougl.), two species common to western North America that are known hosts of mountain pine beetle. In 2013, we harvested two trees of each species on July 29 and 30 and two more on August 5 and 6. In 2014, the same numbers of trees were cut on August 4 and 5 and August 11 and 12, for a total of 48 pines for both years. All trees were free from any sign of damage or disease and approximately 24 cm DBH (diameter at breast height, approximately 1.4m above ground level) (Table 1).

**Table 1:** Mean (SE) diameter at breast height (DBH) (cm) of trees cut (*n* = 4 per species per year) and mean (SE) phloem thickness (mm) of each species for each of 6 logs cut from the bole of each tree. Means within a column followed by the same letter are not significantly different.

	2013		2014	
Species	DBH (SE) cm	Phloem (SE) mm	DBH (SE) cm	Phloem (SE) mm
Ponderosa	25.76 (0.58)	2.02 (0.08)	24.22 (0.21)	3.10 (0.06)a
Lodgepole	24.28 (0.57)	2.01 (0.07)	23.61 (0.48)	3.18 (0.05)a
Jack	22.63 (0.71)	1.56 (0.04)	22.93 (0.55)	2.00 (0.06)c
Red	24.63 (1.30)	1.89 (0.04)	23.10 (0.36)	2.47 (0.04)b
Eastern White	24.60 (0.78)	1.86 (0.06)	24.13 (0.63)	2.50 (0.10)b
Scots	24.03 (0.90)	1.61 (0.03)	23.56 (0.93)	2.09 (0.07)bc
F_5,18_	1.46	2.61	0.83	12.51
*P*	0.25	0.06	0.54	<0.0001

The eastern pines as well as quaking aspen (*Populus tremuloides* Michx.), which served as a negative control for bark acceptance experiments, were sourced, with permission, from the University of Minnesota Cloquet Forestry Center, Cloquet, MN, USA (latitude, longitude: 46.701735, -92.521798). Ponderosa pines were cut with permission from the USDA-Forest Service Rocky Mountain Region from stands in the Black Hills of South Dakota, USA in 2013 (latitude, longitude: 44.12955, -103.48513) and 2014 (44.12587, -103.56700). Lodgepole pines, with permission from the USDA-Forest Service Rocky Mountain Region were harvested from the Bighorn National Forests in the central Bighorn Mountains, Wyoming, USA in 2013 (44.60337, -107.21505 and 44.62710, -107.16303) and 2014 (44.31865, -106.94633 and 44.22341, -106.93212). From each tree, we cut five logs at 1 m lengths and immediately sealed cut ends with paraffin wax to reduce desiccation. Sealed logs were enclosed immediately in black terrapin body bags (BP medical supplies, Brooklyn, NY, USA) to prevent infestation by other insects. We transported all logs to an experiment station in the central Black Hills, SD within 24 h of harvest, and stored them on their cut ends in a closed building until use.

We utilized cut material instead of live trees for several reasons, including regulatory and biosafety concerns in introducing mountain pine beetle and its associated fungi to live trees located outside of its current range. Freshly cut material has often been used by others to assess colonization dynamics of mountain pine beetle [35,42,54,62–66] and allows an assessment of baseline constitutive effects between species in a common garden environment.

### Source of insects

Mountain pine beetles were collected in 12-funnel Lindgren funnel traps [67] with a commercially available pheromone lure (Contech Enterprises Inc, Delta, BC) from 6–8 locations along an approximately 6 km transect during peak flight in the first and second weeks of August in 2013 and 2014 in the central Black Hills National Forest with permission from the USDA-Forest Service Rocky Mountain Region. Collection cups contained clear cellophane shred (Spring-Fill Industries, Northbrook, IL) as refugia to reduce insect damage from crowding. Beetles were collected daily, transferred to Petri dishes lined with a lightly moistened tissue paper (Kimwipe: Kimberly-Clark, Irving, TX), and stored at approximately 5°C. We separated beetles by sex using auditory stridulation within 24 h of trapping [68]. Beetles were stored for 1–5 d before use.

### Experiment 1: Bark entry

To test the frequency at which mountain pine beetles enter the bark of each species of pine, female beetles were caged on logs. Log sections 60 cm in length were cut from the lower bole of harvested trees. We measured the phloem thickness at three equidistant locations around the perimeter of the cut surface, and sealed the cut ends with paraffin wax to reduce desiccation. In 2013, ten 144 cm^2^ cells (12 x 12cm) were constructed around the middle of the log using a border of 32 x 4 mm closed-cell vinyl foam tape (W.J. Dennis & Company, Elgin IL). In 2014, two 625 cm^2^ cells (25 x 25cm) were constructed on each log. Care was taken not to disturb bark texture, and gaps in the cell border were filled with additional strips of tape. We secured charcoal-colored aluminum screening (New York Wire, Hanover, PA) to the cells with staples but ensured no bracing point other than the bark itself was available for beetles to begin boring. We introduced 5 beetles per cell in 2013 and 15 per cell in 2014. Two trees of each species were used in 2013 and three trees in 2014. Scots pine was tested in 2014 but not 2013.

Logs were placed upright indoors with natural light (approx.14L: 10D) and variable temperature (19–24°C). Logs were kept indoors to prevent colonization by other insects during the study. The logs were examined at 24, 48 and 72 h. Bark acceptance by beetles was judged by the presence and color of boring dust (i.e., dark = bark; light = phloem) and visual inspection of whether the insects were visible or had begun vertical boring within the log. At 72 h, we debarked the logs to confirm the number of beetles that had bored through the outer bark. Because no beetles bored into aspen, the negative control, it was not included in statistical analyses. To determine if bark acceptance behavior was similar between cut logs and live trees, we repeated the bark acceptance experiment in 2014 with four live ponderosa pine of similar diameter and origin to the logs used in the laboratory assays.

### Experiment 2: Phloem entry

We examined the propensity of female beetles to initiate tunneling once the phloem was reached. We cut two 40 cm log sections from three of the 1m logs of each tree. In 2013, two additional lengths were cut from each tree for a total of 168 logs in 2013 and 144 in 2014. Phloem thickness was measured, and ends were sealed with paraffin wax, as before. We drilled six equidistant holes that were 5 cm from one cut surface in 2013 and seven holes in 2014. Holes were 63 mm in diameter and just scored the phloem. A female beetle was introduced to each hole within 24–48 h of trees being felled. We placed female beetles in microcentrifuge tubes (0.2 ml capacity; Eppendorf, Hamburg, Germany) with tops removed and inserted the open ends into the holes. Tubes were checked after 12 h for acceptance of phloem. Inactive females that had not entered the phloem (i.e., no boring dust in the microcentrifuge tube) were recorded as rejections. Rejecting beetles were replaced with new females for the following experiments.

### Experiment 3: Brood establishment

We determined the proportion of adult female beetles that established ovipositional galleries and laid fertile eggs after accepting the phloem from 144 (24 of each pine species) of the 168 logs from 2013 and all 144 logs from 2014 used in Experiment 2. Males were added to three holes approximately 18–24 h after the first female introductions. Vinyl screen was loosely attached over the entrance hole to reduce the chance of beetles falling out while the logs were handled. Logs were wrapped in charcoal-colored aluminum screening (New York Wire, Hanover, PA) and secured at both ends with staples to preclude entry by other wood-boring insects or predators before being stored outdoors for autumn and winter of 2013–14 and 2014–15. For a separate experiment [69], a subset of logs were debarked in January of each year. The remaining logs were returned indoors in April and placed in cardboard emergence tubes. Logs were debarked in mid-August after beetles had emerged [69]. To determine brood establishment, galleries of mated females that had been provided a male and had established ovipositional galleries were inspected for a minimum of one horizontal larval gallery.

### Experiment 4: Attraction to tunneling beetles

We assessed differences in attraction of beetles to infested pine substrate in a field study utilizing artificially infested logs in a choice experiment [63]. Twelve sites, no closer than 350 m, and directly adjacent to or within active outbreaks (characterized by pines with fading needles and fresh pitch tubes around the bole) were established in early August of 2013 and 2014 in the Black Hills National Forest with permission from the USDA-Forest Service Rocky Mountain Region. At each location, we arranged seven 12-unit Lindgren funnel traps suspended from iron t-posts spaced every 3 m equidistantly around a ring. The funnel traps were attached to the t-posts with 35.5 cm long aluminum shelving brackets that were secured with wire to the t-posts. Screen logs from experiment two, which contained both paired and unpaired females, were used as bait. One infested log of each of the six pine species was transported to each of twelve field sites within 48–60 h of females being inserted. Logs were arranged at random and fixed with a hook next to each trap with the beetle entry holes midway down the trap length. As a negative control, one trap was left with no log. Traps were checked approximately every 48 h and all insects were removed and counted. Traps remained set for 6 d as beetles are expected to produce relatively consistent amounts of pheromone over this period [70], and were then replaced with a second set of fresh logs that were prepared in the same manner as above. In this manner, approximately 2 wk of data were collected both years, and the signal of tunneling beetles in exposed logs never lasted more than 9 d (i.e., log preparation plus testing time). In 2013, logs were kept on the same t-post at each site for the length of the week, so total beetle catch for that treatment was summed for each week. In 2014, logs were re-randomized at each site each time a collection occurred. In 2013, twelve sites were used each week. In 2014, twelve sites were used the first week, and six sites the second week.

### Chemical analysis of tree material

We collected phloem samples from logs to quantify monoterpene concentrations. A bark sample (approx. 5x5 cm) with the phloem intact was collected from a log of each of the four trees of each species in 2014 and two trees of red, eastern white, jack and ponderosa pine and one tree of lodgepole and Scots pine in 2013. Samples were removed within four days after trees were cut and stored in a freezer at -20°C until processing. A 1.5 cm^2^ phloem sample was removed from the bark and phloem sample and cut into approximately 1 mm^2^ pieces. Phloem constituents were extracted twice with 0.75 mL (1.5 mL total) high performance liquid chromatography grade hexane for 24 hours in a 2 mL vile at room temperature. Hexane was removed from the sample after each extraction with a 1 mL syringe. The two extractions were combined and passed through a 0.45 μm polyvinylidene fluoride syringe filter (Analytical Sales and Services Inc, Pompton Plains, NJ) in preparation for gas chromatography mass spectrometry (GCMS) analysis. The extracted phloem was placed in a fume hood for 1 wk at room temperature to dry. Once dry, the mass of phloem was recorded and used to normalize the concentrations of organic extracts.

GCMS analysis was carried out by using a Shimadzu QP2010S equipped with a Restek Rxi-5 ms column (30 m x 0.25 mm). Helium was used as the carrier gas at a column flow rate of 0.60 mL/min. Initial oven temperature was 55°C. This temperature was held for 5 min., stepped to 70°C at 1°C per minute, and then stepped to 160°C at 15°C per minute and held for 2 minutes. Finally, the oven was stepped to 250°C at 30°C per min. and held for 4 minutes.

All samples and standards contained helptyl acetate as an internal standard at a final concentration of 0.025 mM. Analytic standards of the phenylpropanoid 4-allylanisole and the most common and biologically important monoterpenes [71] α-pinene, β-pinene, 3-carene, myrcene, limonene, and camphene were used to generate calibration curves and response factors compared to the internal standard. β-phellandrene, also an important monoterpene for which a standard was unavailable, was identified as a 99% match with the NIST08 library. These titration curves and response factors were used to determine final concentrations and ratios for each compound in the phloem extracts. β-phellandrene and limonene co-eluted under the separation conditions. The concentration of β-phellandrene was approximated by subtracting the limonene signal based on unique ions in the mass spectrum and its calibration curve. The remaining peak area was attributed to β-phellandrene and used to approximate its concentration.

### Analysis

Statistical analyses were completed in mixed effects frameworks in R (R Core Team, 2014). Separate generalized linear models with binomial distributions (lme4 package in R) were used to model the proportions of beetles of the total exposed to the treatment that entered the bark, accepted the phloem, and established brood, respectively. Fixed effects in the model were tree species, tree origin (historical or novel host), phloem thickness, and total monoterpene concentrations. Random effects in the analysis of data from Experiments 2–4 include tree and log nested within tree. In selecting the most parsimonious variables that could explain each response variable examined, we relied on graphical data analysis, Akaike’s Information Criteria to judge model suitability (AIC), and p-values associated with inferential tests of the significance of the variables (α = 0.05).

We examined how phloem thickness, individual monoterpene concentrations and the number of mountain pine beetles captured in funnel traps (Experiment 4) varied with pine species in separate mixed-effect analysis of variance (ANOVA) models. Site and week were included as random effects. To meet model assumptions of homoscedasticity and normality of errors, all trap data and concentrations of 4-allylanisole were square-root transformed, and concentrations of β-pinene, 3-carene, myrcene, limonene, β-phelandrene and camphene were log(y+1) transformed. Where significant treatment effects existed (α = 0.05), protected least significant difference tests were used to separate means in multiple comparisons [72].

We tested outliers by examining whether the presence of suspicious data points statistically changed the magnitude of the effect of interest (e.g., species of pine) on the response variable (e.g., number of insects captured). We did this by including a binomial indicator variable for suspiciously high trap catches as a covariate in the mixed effects model. If the P-value associated with the questionable catch was less than 0.05 divided by the total number of observations (i.e., Bonferroni’s correction), it was considered an outlier. No outliers were found in 2013; however, in 2014, three points were removed. Two of these points came from a trap near a newly-attacked tree, which can skew catch numbers [65].

We constructed a test statistic to assess the degree of similarity between the rankings of pine species used as baits to capture flying beetles in Experiment 4 in 2013 and 2014. Pine species both years were ranked from most attractive to least attractive based on mean numbers of insects captured. The test statistic was devised by squaring the differences in ranks per treatment between years and summing those values. This procedure was then repeated 999 times with randomly generated rankings for both years. The placement of the test statistic from the empirical data relative to the 999 randomly generated test statistics reflects the probability of rankings having the same degree of similarity between years.

A Monte Carlo simulation was used to obtain an integrated estimate of the probability of brood production by a female alighting on each pine based on the outcomes of Experiments 1–3. Maximum likelihood estimates of species-specific proportions of beetles that entered the bark, entered the phloem, and produced brood were integrated into one model. Each parameter was assumed to be normally distributed with mean and variances derived from the maximum likelihood estimates of the logit-linked transformed proportions. A random draw was taken from each of the three distributions, then multiplied to obtain an estimate of susceptibility for a given species. The model was run 100,000 times for each pine species to obtain an overall susceptibility distribution. The upper and lower 2.5 percentiles of the distribution were truncated to obtain the middle 95% of the distribution, indicating susceptibility of a given pine species.

## Results

### Experiment 1: Bark entry

Overall, 532 of the 840 females initiated boring within 72 h of assay initiation, a boring rate of 63.3%. Beetles bored into the bark of all species of pines tested although the cumulative proportion that entered the bark by 24, 48 and 72 h varied among species (Table 2). Approximately 20–25% more beetles entered pines representing their historical hosts than novel eastern hosts by the 2-day and 3-day time points (contrasts; Day 1: χ^2^ = 1.68, df = 1, *P* = 0.20; Day 2: χ^2^ = 7.67, df = 1, *P* = 0.006; Day 3: χ^2^ = 7.80, df = 1, *P* = 0.005). Phloem thickness did not affect propensity of an insect to penetrate the bark (Day 1: χ^2^ = 0.57, df = 1, *P* = 0.45; Day 2: χ^2^ = 2.82, df = 1, *P* = 0.093; Day 3: χ^2^ = 1.60, df = 1, *P* = 0.21).

**Table 2:** Proportion of female mountain pine beetles that bored through the bark of six species of pine over a three day period. Means within a column followed by the same letter are not significantly different.

		24 hours	48 hours	72 hours
Species	Host	mean % (±95%CI)	mean % (±95%CI)	mean % (±95%CI)
Ponderosa	historical	44.0 (36.3, 52.0) a	67.3 (59.4, 74.4) a	74.8 (66.5, 81.7) a
Lodgepole	historical	28.0 (19.9, 36.9) b	58.7 (50.6, 66.3) ab	68.8 (60.0, 76.4) ab
Jack	novel	31.3 (23.0, 40.7) b	50.7 (42.7, 58.6) b	56.7 (47.7, 65.3) bc
Red	novel	38.7 (29.5, 48.4) ab	60.0 (52.0, 67.5) ab	68.1 (59.3, 75.8) ab
Eastern White	novel	17.3 (11.2, 24.9) c	28.7 (22.0, 36.4) c	47.3 (38.5, 56.3) c
Scots	novel	30.0 (19.7, 42.1) b	57.8 (47.4, 67.5) ab	65.7 (54.1, 75.7) ab
χ^2^		20.68	50.36	24.55
*P*		0.00093	1.168E-09	0.00017

No beetles bored into aspen, the negative control, which is therefore excluded from the analysis.

**Fig 2:**
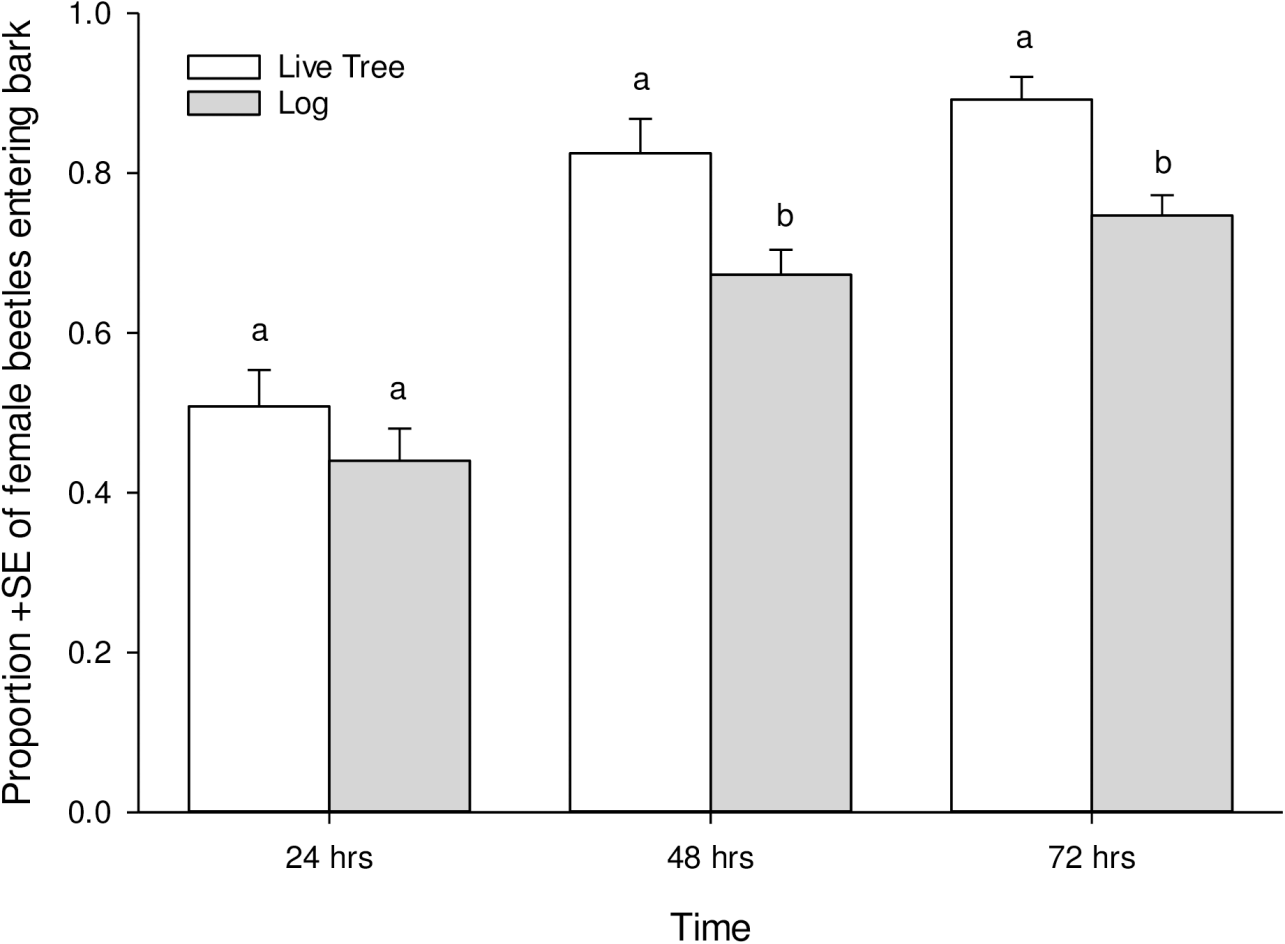
Comparison of the mean (+SE) proportion of female beetles entering four live ponderosa pine trees (*n* = 120 beetles) and logs cut from fiveponderosa pine trees (*n* = 150 beetles) over a three day period. Bars within a time period with the same letter are not significantly different.

When we compared our laboratory assays to insects boring in live trees, we found that the proportion of beetles entering the bark of live trees versus cut logs of ponderosa pine were similar after 24 h (Fig 2; χ^2^ = 0.88, df = 1, *P* = 0.35). However, approximately 10% more insects entered the live trees versus cut logs after 48 h (χ^2^ = 7.32, df = 1, *P* = 0.007) and 72 h (χ^2^ = 6.38, df = 1, *P* = 0.01). Overall success rates approached 95% for the live trees, and 80% for the cut logs (Fig 2). No beetles initiated boring before subsequently rejecting the live hosts in the three days of observation.

### Experiment 2: Phloem entry

**Fig 3:**
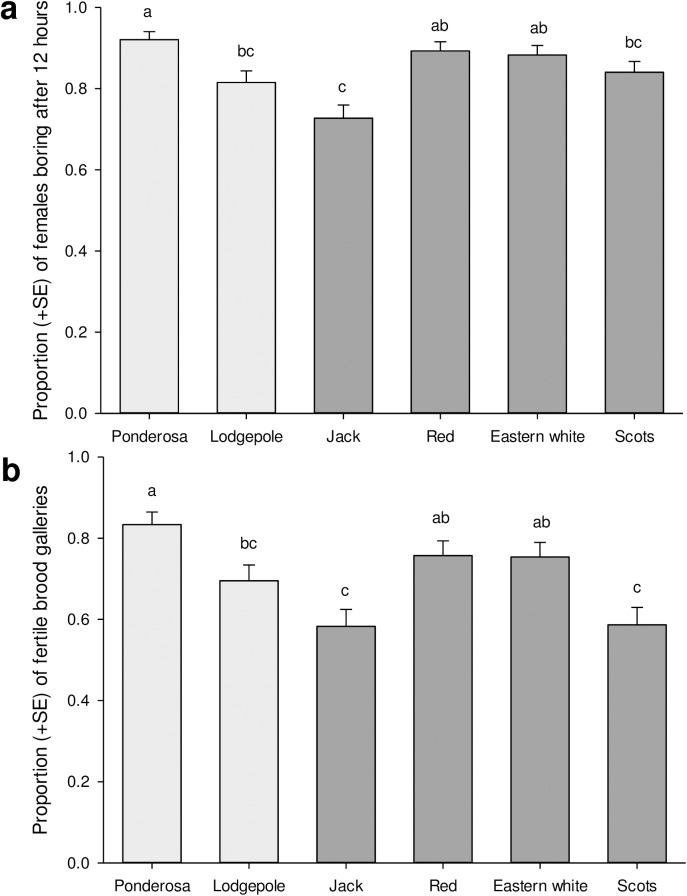
Mean (+SE) phloem acceptance and egg gallery establishment. (A) Mean (+SE) proportion of female beetles accepting phloem after 12 hours when placed into direct contact with phloem through pre-drilled holes. (B) Mean (±SE) proportion of galleries from logs infested with mountain pine beetle with larval galleries present after at least five months. Presence of larval galleries indicates mated pairs accepted the log and laid fertile eggs. Light and dark bars represent historical and novel pine hosts respectively. Bars with the same letter are not significantly different.

Our second experiment assessed whether beetles that had entered the bark would subsequently tunnel into the phloem. The majority (84.7%) of the 1123 beetles in this experiment actively bored into the phloem within 12 h of being introduced to the phloem, although the proportion that bored varied by species (Fig 3A; χ^2^ = 19.12, df = 5, *P* = 0.002). Jack pine exhibited the lowest percentage of females entering the phloem, with 15–19 percent fewer beetles entering jack pine phloem than ponderosa, red or eastern white pine phloem (Fig 3A). There was no overall effect of historical association of pine with the mountain pine beetle on phloem entry (χ^2^ = 0.907, df = 1, *P* = 0.34). Likewise, we found no overall effect across years of phloem thickness on the proportion of females that entered the phloem (χ^2^ = 0.027, df = 1, *P* = 0.87). However, there was a weak negative effect of phloem thickness on likelihood of phloem entry in 2014 (χ^2^ = 4.90, df = 1, *P* = 0.027). There was no relationship between total monoterpene concentration and insect’s acceptance of phloem (χ^2^ = 0.15, df = 1, *P* = 0.70).

### Experiment 3: Brood establishment

A total of 840 galleries were established by the paired female and male beetles from the subset of logs used in Experiment 2. Of these galleries, 70.2% established brood, although the likelihood of brood establishment varied among pine species (Fig 3B; χ^2^ = 27.18, df = 5, *P* < 0.0001). The percentage of females that established brood was 13% greater in historical hosts than in novel hosts (χ^2^ = 4.70, df = 1, *P* = 0.03). Brood establishment rates appear to be driven, in part, by phloem thickness, with thicker phloem in historical hosts (Table 1). While there was some evidence for a relationship between likelihood of brood establishment and phloem thickness overall (χ^2^ = 3.74, df = 1, *P* = 0.053), we found that phloem thickness explained more variation in likelihood of successful brood establishment in 2014 than did species of pine. Thinner phloem (χ^2^ = 22.4, df = 1, *P* < 0.0001) and greater total monoterpene concentrations (χ^2^ = 6.1, df = 1, *P* = 0.01) resulted in fewer successful galleries.

### Modeled susceptibility to colonization

**Fig 4:**
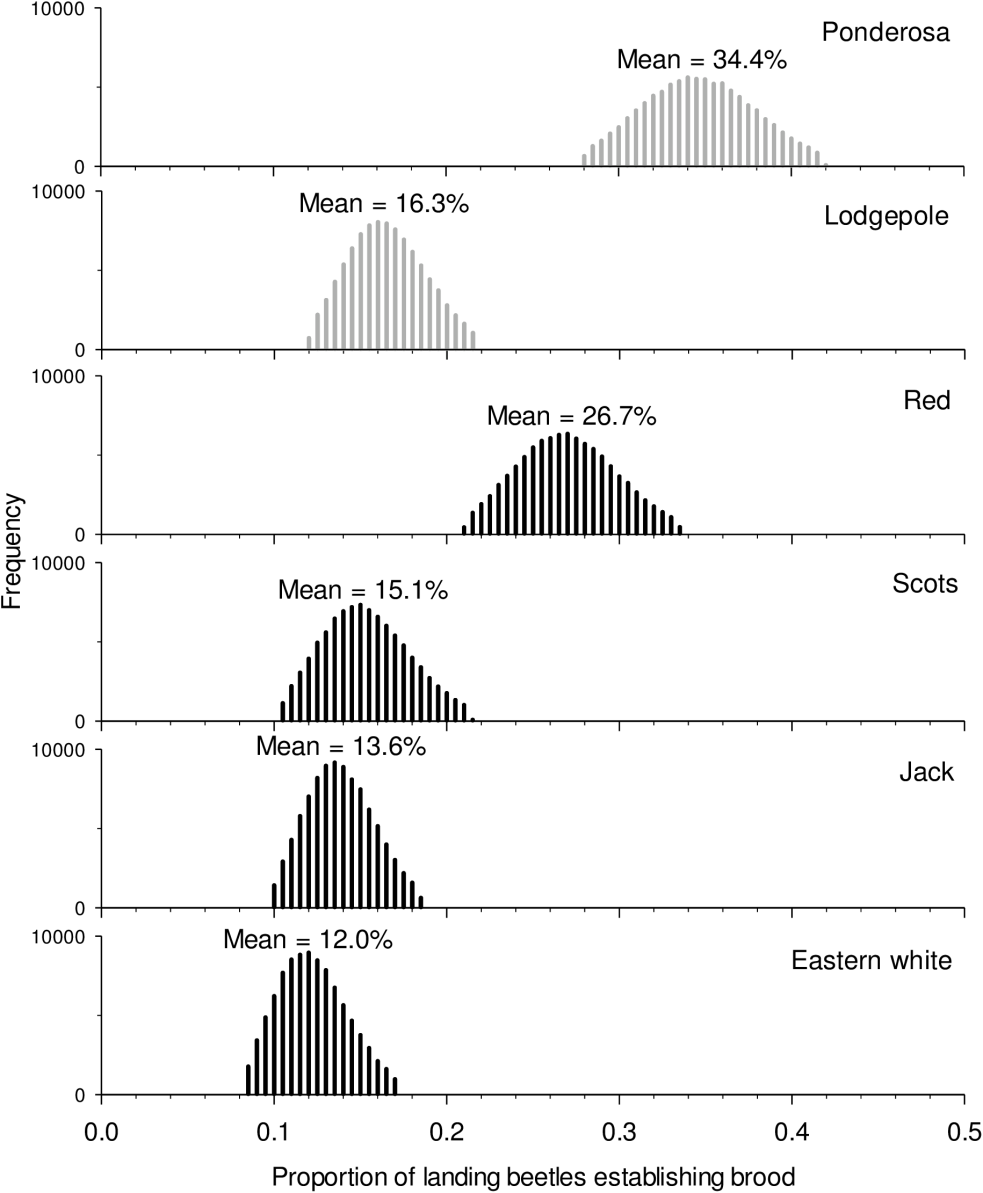
Monte Carlo simulation integrating bark entry, phloem acceptance and egg gallery establishment for cumulative susceptibility. Percentages indicated the likelihood of a landing beetle establishing a fertile egg gallery. Light and dark bars represent historical and novel hosts respectively. Probability distributions show the middle 95% of the distribution, with upper and lower 2.5% of the tails removed.

By integrating the results of Experiments 1–3 (i.e., bark is entered, boring is initiated in the phloem, and brood establishment occurs), we examined overall susceptibility to colonization (Fig 4). In general, less than 50% of adult females placed on the bark completed the series of discrete steps in host colonization that would result in live progeny under the bark. There were notable differences between species, however. Ponderosa pine appeared to be twice as susceptible to mountain pine beetle colonization as lodgepole pine. Susceptibility also differed between novel hosts, with red pine being more susceptible than any of the other novel hosts and even lodgepole pine. Eastern white pine was the least susceptible pine, although still similar overall to lodgepole pine (Fig 4).

### Host attraction

**Fig 5:**
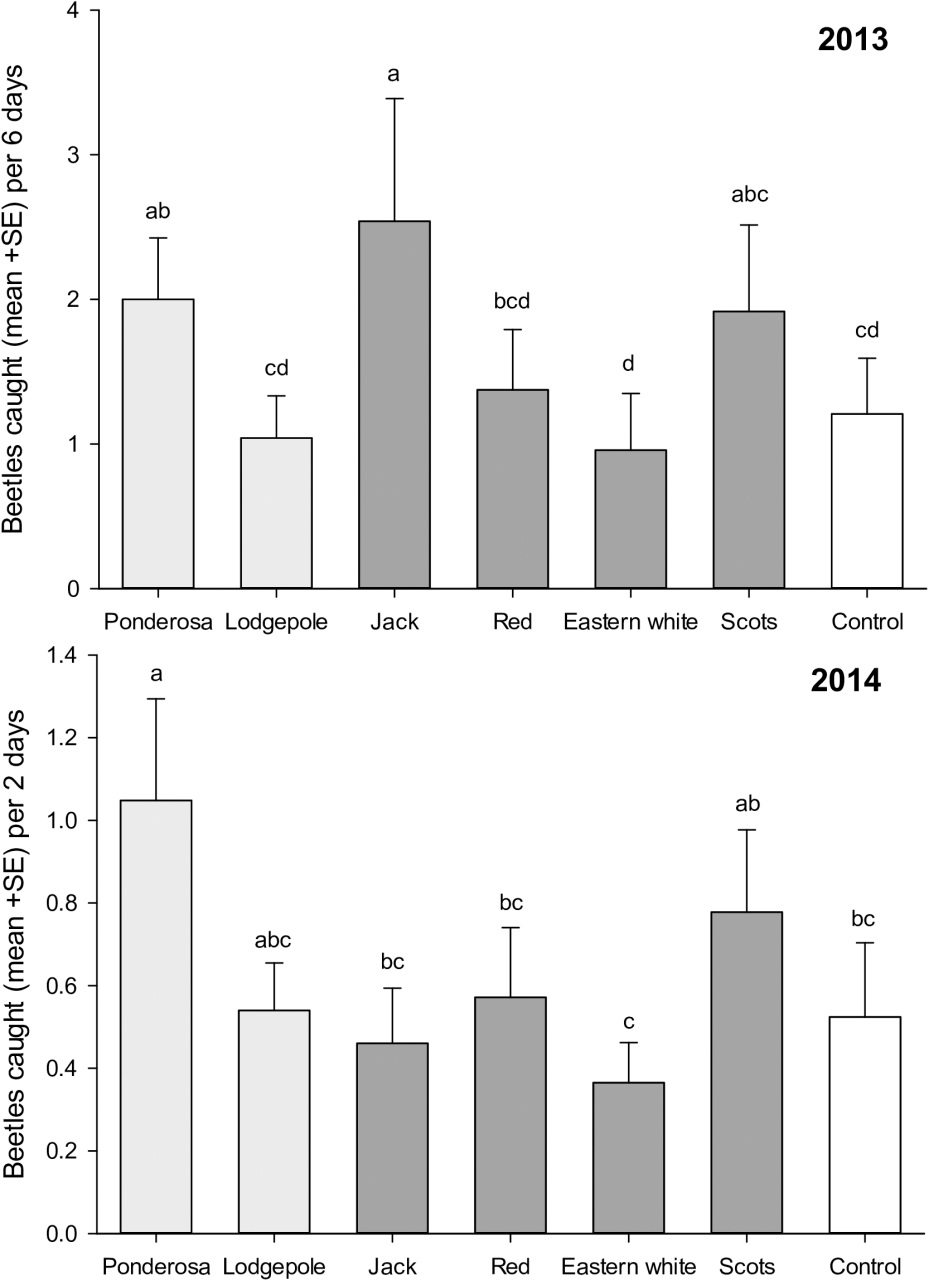
Mean (+SE) number of beetles caught in funnel traps. Traps were adjacent to various species of logs infested with boring beetles (*n* = 12 sites) over 2013 and 2014 flight periods. Light grey and dark grey bars differentiate historical and novel hosts respectively. White bars represent the control. Bars with the same letter are not significantly different.

The number of mountain pine beetles caught in funnel traps associated with infested logs varied among pine species in 2013 (F_6,138_ = 3.70, *P* = 0.002) and 2014 (F_6,372_ = 3.10, *P* = 0.009) (Fig 5). In general, traps associated with ponderosa and Scots pine logs caught the most beetles, while traps associated with logs of eastern white pine captured the least. The rankings of attraction between the seven treatments were consistent between years, with the exception of jack pine. Traps baited with infested jack pine captured more beetles than ponderosa pine in 2013, but fewer than all but eastern white pine in 2014. While there was not a significant likelihood (*P* = 0.17) of rankings being consistent between years with jack pine included, removal of the jack pine treatment resulted in a significant likelihood of consistency of the remaining six rankings between years not being due to chance (*P* = 0.006). Despite the consistency of these patterns, overall, very few beetles were captured. Traps associated with infested ponderosa pine logs were the only treatments that caught significantly more beetles in both years than unbaited control traps (Fig 5). More flying beetles were caught in traps associated with historical than novel hosts in 2014 (F_2,376_ = 4.41, *P* = 0.013), although this pattern was not apparent in 2013 (F_2,142_ = 0.595, *P* = 0.553).

### Chemical concentrations in pines

**Fig 6:**
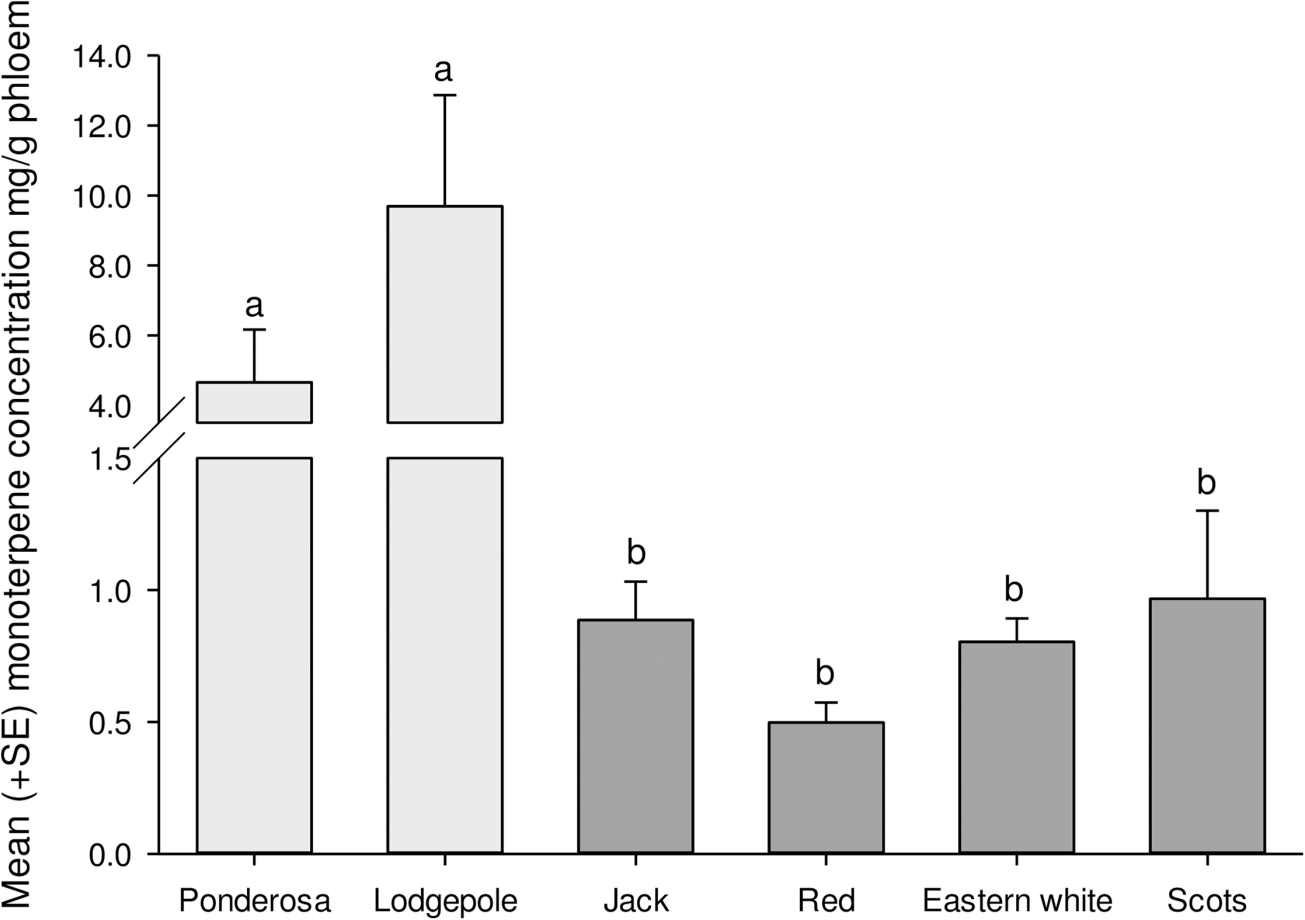
Total mean (+SE) monoterpene concentration (mg/g of phloem) of six species of pines used in this study. Samples were extracted from two uninfested logs of each pine within four days of being cut in 2013 with the exception of Scots and lodgepole from which only one was taken, and each of the four trees of each species in 2014. Light and dark bars represent historical and novel hosts respectively. Bars with the same letter are not significantly different.

**Fig 7:**
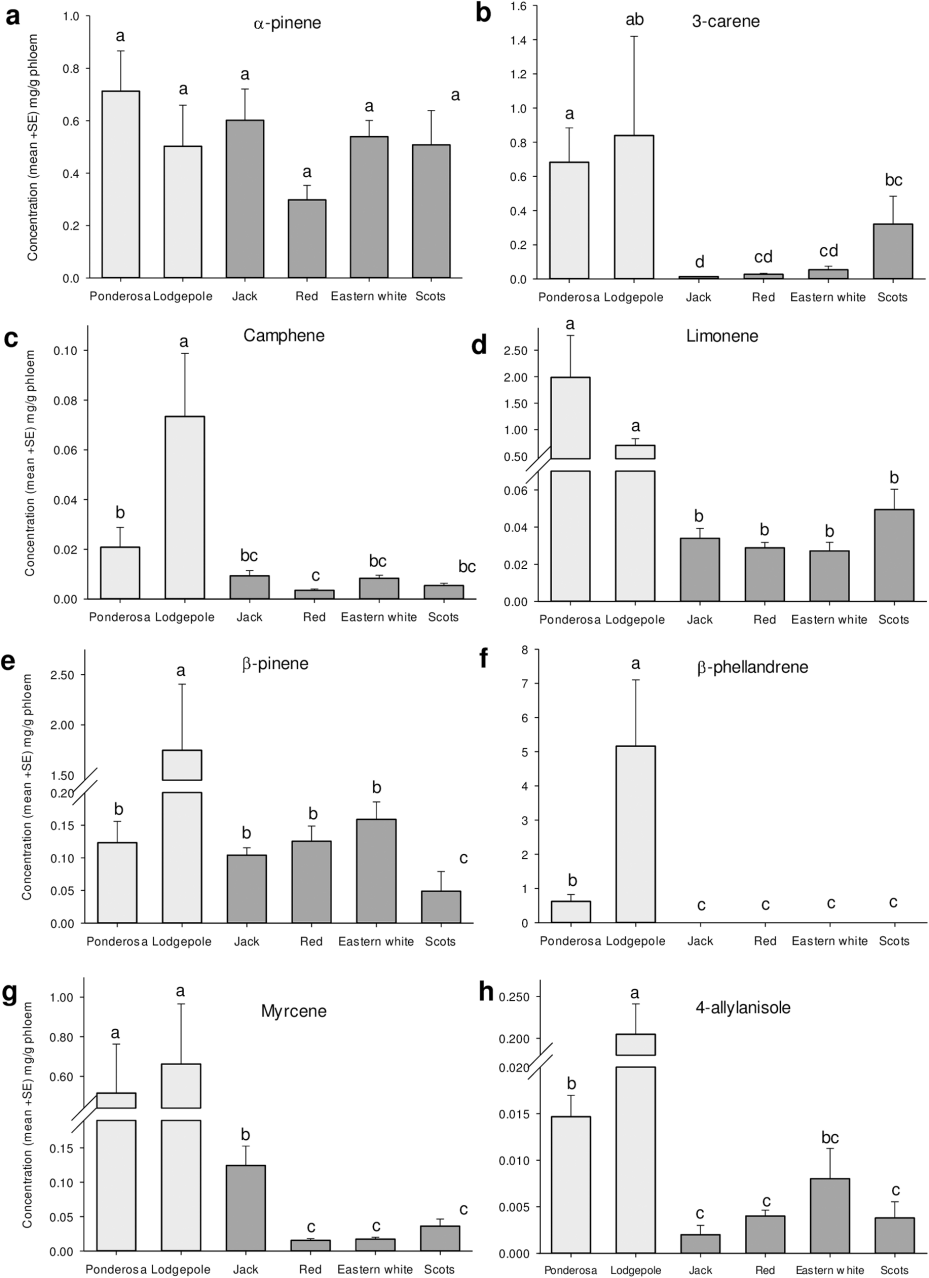
Mean (+SE) absolute chemical composition of logs from the six species of pines used in this study. Samples were extracted from two logs of each pine within four days of being cut in 2013 with the exception of Scots and lodgepole from which only one was taken, and each of the four trees of each species in 2014. Light and dark bars represent historical and novel hosts respectively. Bars with the same letter are not significantly different. Note that scales of *y*-axis vary between chemicals.

**Fig 8:**
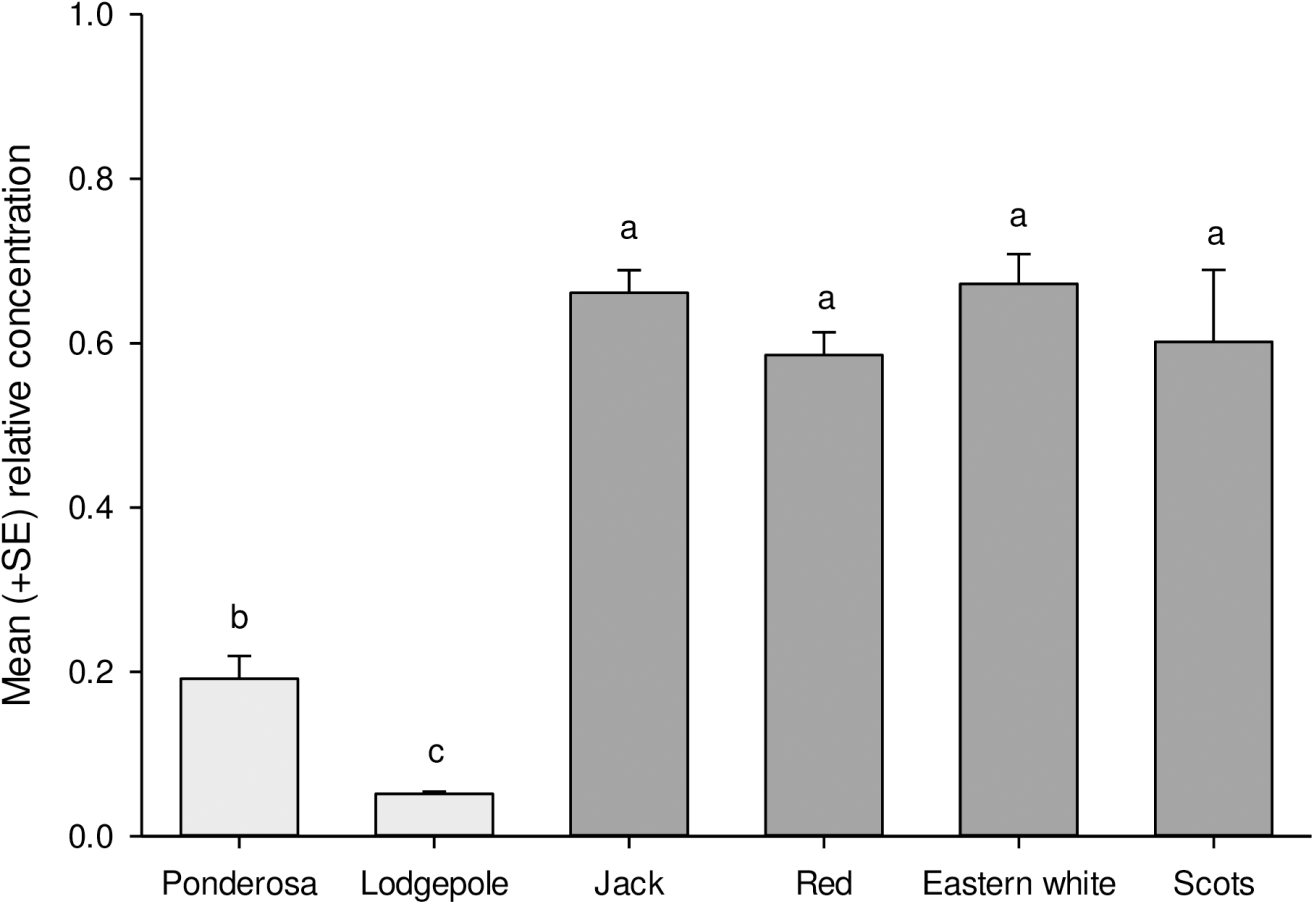
Mean (+SE) relative concentration of α-pinene in pine logs in relation to the seven primary monoterpenes measured. Samples were extracted from two logs of each pine within four days of being cut in 2013 with the exception of Scots and lodgepole from which only one was taken, and each of the four trees of each species in 2014. Light and dark bars represent historical and novel hosts respectively. Bars with the same letter are not significantly different.

The total absolute concentration of key monoterpene of known biological significance, present in the phloem, differed among pine species (Fig 6; F_5,28_ = 12.91, *P* < 0.0001). On average, historical hosts (i.e., ponderosa and lodgepole pines) had 6 and 8 times more total monoterpenes than did novel hosts on average, respectively. Notably, Scots pine was the only novel host to have absolute concentrations of a known pheromone synergist, 3-carene, similar to those of historical pines (Fig 7B). We found minimal concentrations of limonene and the phenylpropanoid 4-allylanisole, two known beetle deterrents, in each species of novel hosts, including red pine. The only chemical that did not differ among pines in absolute concentrations was α-pinene (Fig 7; F_5,28_ = 1.44, *P* < 0.24), though relative concentrations of α-pinene (i.e., percent α-pinene relative to all other monoterpenes measured) did vary (Fig 8; F_5,28_ = 42.1, *P* < 0.0001).

## Discussion

Our results fail to provide evidence that constitutive physical or chemical defenses of novel hosts *a priori* protect pine species from an aggressive herbivore by interfering with discrete colonization behaviors. Constitutive monoterpene concentrations can be lethal to other bark beetles in just a few days [58], or deter mountain pine beetle attacks altogether [59,73]. Induced defenses are also critical to tree defense from these insects [45,74], but are only stimulated after the insects and their fungi have breached the host [62,75,76]. Indeed, we found that entry rates of female beetles in our freshly-cut logs and live trees were similar after 24 h (Fig 2). Moreover, our findings that 68.7% of beetles entered the bark of logs of lodgepole pines after three days are similar to boring rates of 61.9% of beetles on live lodgepole pines over three days in Alberta, Canada [77]. Thus, even though inducible defenses would presumably increase upon beetle/fungal challenge in live novel hosts (impossible to test at this time due to quarantine regulations), we expect that our results are comparable to what might occur on live trees in early stages of attack, the focus of this behavioral study.

The consistent pattern of attraction to infested logs of different species between years (*P* = 0.006), and few treatments being more or less attractive than the control (Fig 5), is consistent with certain aspects of their chemical profiles. For example, the concentration of *trans*-verbenol released by boring beetles is correlated with the amount of its monoterpene precursor, α-pinene, present in the phloem [70]. In our study, we note that attractive ponderosa (2013, 2014) and jack pines (2013; Fig 7A) also tended to exhibit higher absolute concentrations of α-pinene than other species, although the mean absolute concentration of α-pinene was statistically similar across species (Fig 7A). Relative, rather than absolute, α-pinene concentration has also been suggested to be important in explaining differential beetle attraction [65]. However, while red, eastern white, and Scots pines exhibited 3.5–4.5 times the relative proportions of α-pinene vs. ponderosa pines (Fig 8), none were more attractive, suggesting that concentrations of other volatile monoterpenes that enhance attraction to mountain pine beetle pheromones may explain observed differences in attraction.

Concentrations of synergists may offer further explanation. Reduced concentrations of synergists would result in low attraction even if high concentrations of *trans*-verbenol were produced [39,78]. Indeed, all four novel host candidates had significantly lower concentrations of myrcene [78,79] than lodgepole and ponderosa pines, and all but Scots pine had lower concentrations of 3-carene [42] than the historical hosts (Fig 7). Terpinolene has also been show to synergize response of flying beetles to *trans*-verbenol [78]. Terpinolene is present in high concentrations in ponderosa, lodgepole and Scots pine [42,46,80,81] but at low concentrations or absent in jack, red and eastern white pine [66,70,82].

Greater concentrations in lodgepole pine of 4-allylanisole (Fig 7H), a phenylpropanoid deterrent of mountain pine beetle and other bark beetles may explain why our lodgepole pine logs were less attractive than ponderosa pine logs [46,83–85]. Possible deterrence to flying beetles by 4-allylanisole suggests a defensive adaptation in lodgepole pine to historical beetle pressure not strongly developed in novel hosts, and deserves further study. The hypothesis that 4-allylanisole has evolved as a deterrent is further supported by the fact that ponderosa and eastern white pine, the species with the second and third highest concentrations of 4-allylanisole respectively (Fig 7H), have also historically faced the two other most aggressive bark beetles in the *Dendroctonus* genus [86,87]; western pine beetle (*D*. *brevicomis*) and southern pine beetle (*D*. *frontalis*), respectively, and are also likely deterred by this chemical [83,85].

### Colonization behaviors in novel northeastern hosts

Our results suggest that eastern forests are likely susceptible to the mountain pine beetle due to a general fit between historical and novel host traits and beetle behaviors [9]. By integrating the three steps in host acceptance, once attraction has occurred, we were able to estimate overall susceptibility to compare beetle preference in historical and novel hosts (Fig 4). Susceptibility of Scots, jack and eastern white pine was similar to lodgepole pine, and red pine was similar to ponderosa pine. Here, we provide a summary of fit for each “novel” species based on beetle behaviors and chemical traits in our experiments.

Overall, the least susceptible novel host, eastern white pine, was no more susceptible to mountain pine beetle than the least susceptible historical host, lodgepole pine (Fig 4). Some resistance to mountain pine beetle in lodgepole pine was evident at each stage of colonization. In contrast, the constitutive resistance displayed in eastern white pine is primarily conferred at the bark level as less than 50% of the beetles had entered the phloem of eastern white pine even after three days exposure (Table 2). Bark rejection may be due to physical traits such as bark texture [88,89], high lignin content [90], or repulsive gustatory cues [35,91]. Resistance conferred by the bark of eastern white pine may reduce its susceptibility at low beetle densities, but does not imply that the pine will not be attacked, or that stands will fully resist mountain pine beetle populations. Indeed, a high proportion of the eastern white pines that beetles entered were successfully attacked and killed in an arboreta in Idaho in the 1960s [61] and 2014 [69]. Primary reliance on resistance at only one point in the colonization process suggests that once the bark is breached, mountain pine beetle will demonstrate little further deterrence. This corroborates reports of general resistance by eastern white pine to southern pine beetle at endemic levels, but heightened susceptibility at outbreak levels when few other options were available [92,93]. We do further note that eastern white pine was the least attractive to foraging beetles (Fig 5), likely due to low concentrations of synergists and possibly higher concentrations of 4-allylanisole (Fig 7).

Overall, red pine appears to exhibit the highest suitability for mountain pine beetle of the novel hosts examined (Fig 4). Beetles tunneled into the bark of red pine at rates greater than eastern white pine (Table 2), although infested red pine logs were no more attractive than controls to foraging beetles (Fig 5). Consistent patterns of reduced attraction in our study likely reflects low concentrations of pheromone synergists in red pine phloem (Fig 7), supporting work by others who also found low concentrations of pheromone synergists but yet also demonstrated pheromone production by mountain pine beetle in red pine logs[66]. Induced defenses of live red pines in response to fungi vectored by the beetles could also reduce overall susceptibility. In general, red pine demonstrates rapid and high induced monoterpene responses to pathogenic fungi [58,94]. Induced responses to *Grosmania clavigera* and *Ophiostoma montium*, the common fungal associates of mountain pine beetle [95], remain unquantified, and induced responses can differ between fungal species [58,75,96]. Regardless, attacks on red pines in an arboreta in Idaho have demonstrated apparent susceptibility [61].

Infested Scots pine, and possibly jack pine, may be more attractive to flying beetles due to the presence of pheromone synergists in their phloem. Jack pine in Alberta have high concentrations of the synergist 3-carene [70,97] and are particularly attractive to mountain pine beetles [42]. However, eastern jack pine populations have little of this monoterpene [70], although it does have the greatest concentration of another synergist, myrcene, among the novel hosts (Fig 7). Increased relative attraction of mountain pine beetle to infested Scots pine may be due to higher concentrations of 3-carene and terpinolene [80] relative to the other novel hosts. This greater attraction may also explain why Scots pines were the only common northeastern pine attacked in an arboretum in California [60] and why they were the first trees to be attacked in the recent attacks at Shattuck Arboretum in Idaho [69].

It is unclear why attraction to traps associated with infested jack pine varied between years while ranking of attraction to other host treatments remained remarkably consistent (Fig 5). Possible reasons for variation in attraction may include phloem thickness, age, abiotic variation between years or variation in chemotypes. Experimental design precludes us from assessing within-species variation in the present work, but variables affecting variation in insect attraction within a host species merits further study, and has been suggested for jack pine previously [70,98].

Reduced susceptibility in Scots and jack pine relative to the highly susceptible red pine (Fig 4) may be correlated with thinner phloem (Table 1) as previous observations in arboreta where nearly half [61] or all [60] attacks on live Scots pines were unsuccessful. Phloem thickness is positively correlated with attack probability [99] and reproductive success in mountain pine beetle [100–103], although its relationship with colonization success has been less well studied. A positive correlation between phloem thickness and colonization success supports the preference-performance hypothesis, which posits that parents choose the most suitable host for offspring fitness [104,105]. Since thin phloem results in fewer offspring [102], preference for pine species with thick phloem once the outer bark has been breached suggests that female assessment of phloem thickness at early stages of colonization drive this preference. Phloem thickness is a plastic trait that may vary between years. Indeed, we observed generally thicker phloem the second year. Phloem thickness is positively related to growth rate and tree diameter [106,107], although overall factors affecting phloem thickness deserve more study. Reduced phloem thickness at older ages is consistent with greater stand susceptibility in over mature stands [12]. Particularly high resin flow in live Scots pine [108], relative to lodgepole pine [109], may also provide additional defensive capabilities in this host that we did not test here.

### Conclusions

Our study is the first to quantify how the initial colonization behaviors of mountain pine beetle vary among historical and novel hosts in a common garden environment. We found little evidence that constitutive defenses, critical in early stages of attack, will preclude mountain pine beetle from colonizing eastern pines. Red pine may be most susceptible to landing beetles while eastern white pine, similar to observations with southern pine beetle in the southeastern United States [92,93], may be least susceptible.

We do note that colonization (i.e., susceptibility) is distinct from reproduction (i.e., suitability), which was not the focus of this study. Tree mortality can occur after a colonization event, irrespective of the successful reproduction of the insect progeny however. Mountain pine beetle, like some other bark beetles, vector virulent fungi [110] that extract nutrients from the sapwood [111] and reduce water flow from the roots to the canopy, accelerating tree mortality [112,113].

Our results may be useful and applicable to other systems undergoing dramatic range shifts. While the mountain pine beetle is a future threat to common northeastern pines that have never exhibited association with an aggressive bark beetle, the southern pine beetle has already begun expanding its range north from the southeastern United States [114]. This insect has spread hundreds of miles north in recent years and was found in New England in 2014 [115], where it has successfully attacked red, eastern white and Scots pines (Dodds, K. pers. comm.). Indeed, both the mountain pine beetle and southern pine beetle attack trees in a similar fashion via mass attacks, and respond in similar ways to host monoterpenes during colonization events [71].

Much future work is necessary to more fully understand the impacts mountain pine beetle may have among novel hosts as the insect moves higher in elevation and expands eastward, subjecting new pine populations to attack [24,86]. Future work should investigate foliar volatile organic compounds and their potential role in colonization [73], suitability of eastern pines for fungal and microbial symbionts, differences in physical and induced defenses of these pines to beetle-vectored fungi, novel interactions with other subcortical insects and predators, and reproductive potential. Our finding that novel northeastern pines have little innate defenses that preclude susceptibility to beetle colonization provides further evidence that accidental introduction or continued range expansion into eastern areas of North America could have serious effects on several species of economically and ecologically important native pines [116].
